# Mechanistic investigation of clarithromycin in ulcerative colitis involving Kv1.3 downregulation and modulation of the Th17/Treg balance

**DOI:** 10.3389/fimmu.2026.1957785

**Published:** 2026-10-20

**Authors:** Jiaying Wu, Wen Zhang, Xuwen Mao

**Affiliations:** 1 College of Pharmacy, Xinjiang Medical University, Urumqi, China; 2 Xinjiang Key Laboratory of Natural Medicines Active Components and Drug Release Technology, Xinjiang Medical University, Urumqi, China; 3 Xinjiang Key Laboratory of Biopharmaceuticals and Medical Devices, Xinjiang Medical University, Urumqi, China; 4 Charles E. Schmidt College of Science, Florida Atlantic University, Boca Raton, FL, United States

**Keywords:** clarithromycin, immune regulation, Kv1.3 channel, molecular docking, ulcerative colitis

## Abstract

**Introduction:**

Present therapies for ulcerative colitis (UC) frequently encounter challenges due to medication resistance and side effects, underscoring the necessity to discover secure and efficacious therapeutic targets. Clarithromycin (CLA), a macrolide antibiotic, has not been fully characterized with respect to its anti-inflammatory mechanisms and therapeutic potential in UC.

**Methods:**

An LPS-induced cellular inflammation model, an LPS-induced acute colitis model in mice, and a DSS-induced rat colitis model were established, with control, model, and CLA-treated groups. The anti-inflammatory, intestinal mucosal-protective, and immunomodulatory effects of CLA were evaluated using CCK-8 assays, ELISA, RT-qPCR, hematoxylin and eosin staining, immunohistochemistry, and flow cytometry. Kv1.3 protein expression was evaluated using immunofluorescence and Western blot techniques, while molecular docking and molecular dynamics simulations were conducted to explore the possible interaction between CLA and Kv1.3.

**Results:**

CLA significantly attenuated the LPS-induced reduction in cell viability *in vitro* and decreased IL-6 and TNF-α levels in cell culture supernatants and mouse serum. *In vivo*, CLA ameliorated elevated disease activity index scores, colon shortening, increased intestinal permeability, and histopathological damage. Immunohistochemistry further showed that CLA reversed the model-associated upregulation of TLR4 and TLR9 and downregulation of Occludin and ZO-1 in colonic tissue. CLA also significantly downregulated Kv1.3 protein expression in colonic tissue. At the same time, computational analyses suggested that CLA could occupy a putative binding pocket within Kv1.3 and maintain a relatively stable binding conformation during the simulation. In addition, CLA was associated with reduced proportions of DCs subset exhibiting maturation- and homing-related phenotypes, correction of the Th17/Treg imbalance, and reduced proportions of effector T cells in colonic tissue.

**Conclusions:**

Clarithromycin significantly ameliorated DSS-induced ulcerative colitis in rats. This therapeutic effect was accompanied by reduced Kv1.3 expression in colonic tissues, suppression of dendritic cell maturation and intestinal homing, correction of the Th17/Treg imbalance, and decreased intestinal infiltration of effector T cells. Molecular docking and molecular dynamics simulations further suggested a potential interaction between clarithromycin and Kv1.3. These findings provide experimental support for the repurposing of clarithromycin in ulcerative colitis and expression-level evidence supporting Kv1.3 as a potential therapeutic target for UC.

## 1 Introduction

Ulcerative colitis (UC) is a persistent, nonspecific inflammatory bowel disorder marked by inflammation predominantly affecting the mucosal layer of the rectum and colon. It exhibits a prolonged, recurrent course, significantly diminishing patients’ quality of life. While the exact cause is not fully understood, ulcerative colitis is typically viewed as resulting from a combination of factors, including genetic predisposition, environmental factors, imbalances in gut flora, and aberrant host immune responses (1). Current clinical treatments for UC mainly include aminosalicylates, glucocorticoids, and biologics; however, their use may be limited by a slow onset of action, secondary loss of response, and an increased risk of infection (2). Accordingly, identifying safe and effective therapeutic agents remains a major research priority.

The disruption of intestinal immune equilibrium, particularly the altered ratio of T helper 17 cells (Th17) to regulatory T cells (Treg), is pivotal in the development of UC. Th17 cells enhance inflammatory responses by secreting pro-inflammatory mediators, including IL-17A and IL-23, while Treg exert immunosuppressive effects by releasing mediators such as IL-10 (3). Moreover, dendritic cells (DCs), essential antigen-presenting cells that link innate and adaptive immunity, directly influence T cell differentiation through their activation status and capacity to migrate to the intestinal mucosa. The voltage-gated potassium channel Kv1.3 is notably abundant in effector memory T cells, macrophages, and DCs. Kv1.3 is crucial for the activation, proliferation, and cytokine production of lymphocytes via influencing membrane potential and calcium signaling (4, 5). Kv1.3 has emerged as a viable therapeutic target for autoimmune disorders, with selective inhibitors showing encouraging efficacy in models of multiple sclerosis and rheumatoid arthritis (6).

In addition to broad-spectrum antibacterial activity, macrolide antibiotics such as clarithromycin (CLA) exert distinct antibacterial-independent anti-inflammatory effects. Clinical evidence indicates that these agents are highly effective in patients with diffuse bronchitis, cystic fibrosis, and certain inflammatory bowel diseases, primarily due to their suppression of nuclear factor-κB (NF-κB) activation and reduction in pro-inflammatory cytokine secretion (7–9). However, the immunomodulatory mechanisms of macrolide antibiotics appear to be heterogeneous: while CLA directly inhibits Kv1.3 channel currents in mouse thymocytes, the structurally similar macrolide azithromycin does not alter these currents and instead modifies the physical properties of the cell membrane by changing membrane capacitance (10). This mechanistic divergence suggests that the immunomodulatory effects of CLA may specifically depend on its direct modulation of the Kv1.3 channel; however, whether CLA exerts therapeutic effects in UC and which mechanisms are involved remain unclear.

Against this background, we established a dextran sulfate sodium (DSS)-induced rat model of colitis. We combined it with *in vitro* cellular experiments and molecular dynamics simulations to systematically investigate the therapeutic effects of CLA in UC and their association with the Kv1.3 channel. We hypothesized that CLA may downregulate Kv1.3 expression and inhibit DCs maturation and homing, thereby modulating the Th17/Treg balance, restoring intestinal immune homeostasis, and alleviating colitis. This study aimed to provide experimental evidence supporting the repurposing of CLA and to explore ion channel modulation as a potential therapeutic approach for UC.

## 2 Materials and methods

### 2.1 Materials

CLA was purchased from Shandong Xinhua Pharmaceutical Co., Ltd. (Lot No. 2301512). DSS, which was used to induce colitis, was obtained from Zhejiang Haizheng Pharmaceutical Co., Ltd. (Lot No. C12750877). A fecal occult blood testing kit for mice was obtained from Shanghai Enzyme-Linked Biotechnology Co., Ltd. (Lot No. 0701A22). The KCNA3 polyclonal antibody (cat. no. 14079-1-AP; dilution, 1:1,000) was purchased from Proteintech (Wuhan, China). The β-actin antibody (cat. no. ab263899; dilution, 1:5,000) was purchased from Beijing Bioss Biotechnology Co., Ltd. (Beijing, China). The standard human colonic epithelial cell line NCM460 was acquired from Shanghai Jining Industrial Co., Ltd. (Catalog No. JN26629). The human colorectal cancer cell line Caco-2 was obtained from Wuhan Punosai Life Technology Co., Ltd. (Catalog No. CL-0050). The IL-6 and TNF-α assay kits (cat. nos. JL14113 and 063016HLP104840701, respectively) were purchased from Shanghai Jianglai Biotechnology Co., Ltd. (Shanghai, China). The NO assay kit (cat. no. BC1475) was purchased from Beijing Solarbio Science & Technology Co., Ltd. (Beijing, China).

### 2.2 Laboratory animals

Male Sprague-Dawley (SD) rats, aged 6 to 8 weeks and weighing approximately 200 ± 20 g, were obtained from the Laboratory Animal Center at Xinjiang Medical University, where they were housed under specific pathogen-free (SPF) conditions. Male C57BL/6J mice, with a weight of 20 ± 2 g, were procured from Weitong Lihua, a laboratory animal vendor based in Beijing (license number SCXK [Jing] 2016-0006). Upon arrival, both species were maintained in the university’s SPF barrier facility registered under SYXK [Xin] 2016-0002. Ambient temperature was maintained at 21 ± 2°C, relative humidity ranged from 40% to 45%, and illumination alternated between 12 h of light and 12 h of darkness. Before any experimental manipulation, the rodents underwent a seven-day adaptation period.

### 2.3 Cell culture and treatment

NCM460 cells were cultured in high-glucose Dulbecco’s Modified Eagle Medium supplemented with 10% fetal bovine serum (FBS) and 1% antibiotic-antimycotic solution. The concentrations of penicillin and streptomycin obtained were 100 U/mL and 100 μg/mL, respectively. Cultures were maintained at 37°C in a 5% CO_2_ environment. To induce an inflammatory response *in vitro*, cells were challenged with lipopolysaccharide (LPS; Sigma, catalog no. L2880) for 24 h. The challenge medium was subsequently removed and replaced with fresh culture medium supplemented with CLA at three concentrations: 1, 10, and 100 μM. Following 24 h of CLA exposure, conditioned media were harvested to assess the anti-inflammatory response. The levels of TNF-α, NO, and IL-6 were then quantified using enzyme-linked immunosorbent assay (ELISA).

### 2.4 Determination of cell viability using the CCK-8 assay

The CCK-8 assay was employed to assess the impact of CLA on the viability of intestinal epithelial cells. NCM460 and Caco-2 cells were introduced into 96-well plates. The cells were initially subjected to LPS (1 μg/mL) for 24 hours, followed by treatment with CLA at final concentrations of 0.01, 0.1, 1, 10, or 100 μM for another 24 hours. Subsequently, 10 μL of CCK-8 reagent was added to each well, and the plates were incubated for 4 hours in a dark setting at 37°C with 5% CO_2_. The optical density at 450 nm was assessed using a microplate reader, with blank medium as the control, and cell viability was subsequently evaluated.

### 2.5 LPS-induced systemic inflammation model in mice

C57BL/6J mice were randomly assigned to six groups (n = 6 per group): Control, Model, dexamethasone (DXM; 4 mg/kg), and low-, medium-, and high-dose clarithromycin (CLA-L, 2 mg/kg; CLA-M, 4 mg/kg; and CLA-H, 8 mg/kg). Control mice had free access to standard chow and water and received no treatment. Mice in the Model, DXM, and CLA groups received intraperitoneal injections of 2 mg/kg LPS once daily for 7 consecutive days and had free access to food. The CLA-L, CLA-M, and CLA-H groups received CLA at 2, 4, and 8 mg/kg, respectively, by oral gavage once daily for 7 consecutive days. The DXM group received dexamethasone at 4 mg/kg by oral gavage on the same schedule. CLA was dissolved in physiological saline for oral gavage, and the vehicle control group received an equal volume of physiological saline.

### 2.6 Induction of chronic colitis

SD rats were randomly assigned to the Control, Model, dexamethasone-positive control (DXM, 4 mg·kg^-^¹), and CLA (4 mg·kg^-^¹) groups, with 10 rats in each group. Rats in the control group were provided with a standard diet and water and received no treatment. A rat model of chronic colitis was induced using a 2% DSS solution in drinking water. The experiment consisted of four 7-day cycles, for a total duration of 28 days. During each cycle, rats were provided with 2% DSS-containing drinking water for the first 5 days, followed by regular drinking water for the subsequent 2 days. CLA (4 mg·kg^-^¹) was administered by oral gavage once daily for 5 consecutive days, beginning on day 1 of each DSS administration cycle.

### 2.7 Assessment of colitis severity and tissue collection

Throughout the experiment, the rats’ body weight, stool consistency, and the presence of fecal blood were systematically observed and documented daily. At the same time, the disease activity index (DAI) was computed using a standardized scoring methodology. At the end of the trial, the rats were compassionately euthanized with an intraperitoneal injection of sodium pentobarbital at a dosage of 180 mg/kg. The colon was meticulously removed in a sterile setting, delicately washed with pre-chilled phosphate-buffered saline (PBS) infused with 1 mM PMSF and 0.2 mM Na_3_VO_4_, and then assessed for dimensions and mass. The colon was longitudinally divided, and a segment of the colonic tissue was swiftly frozen in liquid nitrogen and thereafter preserved at −80 °C until RNA extraction. A separate segment of the intestinal tissue was preserved in 4% paraformaldehyde for future paraffin embedding and histological analysis. Simultaneously, blood specimens were obtained from the abdominal aorta and permitted to coagulate at ambient temperature. The serum was then extracted by centrifugation at 3,500 × g for 15 minutes, portioned into aliquots, and stored frozen. The concentrations of the inflammatory mediators IL-6, TNF-α, and NO in mice were quantitatively assessed using ELISA kits.

### 2.8 Hematoxylin and eosin staining and immunohistochemistry

Colon tissues from all groups were preserved in 4% paraformaldehyde, sequentially dehydrated in ethanol, embedded in paraffin, and sectioned at 5 μm. The sections underwent deparaffinization, rehydration, H&E staining, dehydration, clearing, and mounting. The colon mucosal damage index (CMDI) was scored on a 0–4 scale according to the severity of mucosal structural damage, the extent of crypt loss, epithelial integrity, and the degree of inflammatory cell infiltration. The integrity of colonic mucosa, crypt structure, and infiltration of inflammatory cells were assessed using light microscopy and analyzed by researchers unaware of group assignments.

The sections were deparaffinized, rehydrated, and underwent heat-induced antigen retrieval for immunohistochemistry. Endogenous peroxidase activity and nonspecific binding were inhibited using 3% H_2_O_2_ and blocking techniques, respectively. The samples were incubated overnight at 4°C with primary antibodies against TLR4, TLR9, Occludin (OCC), or ZO-1, followed by a 30-minute incubation at room temperature with the corresponding horseradish peroxidase (HRP)-conjugated secondary antibodies. Immunoreactivity was detected using 3,3′-diaminobenzidine (DAB), while nuclei were stained with hematoxylin. Three non-overlapping fields per section were randomly imaged under identical magnification and exposure settings. Average optical density (AOD) was quantified using ImageJ for semiquantitative analysis, with six independent biological samples per group.

### 2.9 Reverse transcription–quantitative PCR

Total RNA was isolated from cellular and tissue specimens utilizing TRIzol reagent alongside a column-based purification technique. Following the evaluation of RNA purity and concentration with a NanoDrop spectrophotometer, 1 µg of total RNA was converted into cDNA utilizing the PrimeScript™ RT Reagent Kit with gDNA Eraser. The standard two-step PCR amplification program was as follows: Stage 1, initial denaturation at 95 °C for 30 s (1 cycle); Stage 2, 40 cycles of 95 °C for 5 s and 60 °C for 30 s. The total number of amplification cycles was 40, and the reaction volume was 20 μL. The primer sequences used for PCR amplification are listed in **Table 1**. Samples were loaded sequentially in the designated order, and the PCR program was initiated. Upon completion, the PCR data were exported from the instrument. The genes and corresponding experimental groups were selected, and the Ct values were used for subsequent analysis. Relative expression was calculated according to the following equations: ΔCt = target gene Ct value − mean reference gene Ct value; ΔΔCt = ΔCt of the target gene − ΔCt of the target gene in the control group; and the final result was calculated based on 2^-ΔΔCt^.

**Table 1:** Primer sequences used for quantitative real-time PCR.

Gene name	Forward	Reverse
IL-1β	ATGCCTCGTGCTGTCTGACC	TTTGTCGTTGCTTGTCTCTCCTTG
IL-6	CTTCCAGCCAGTTGCCTTCTTG	TGGTCTGTTGTGGGTGGTATCC
IFN-γ	ATGAACGCTACACACTGCATC	CCATCCTTTTGCCAGTTCCTC
IL-12	AGACATCACACGGGACCAAAC	CCAGGCAACTCTCGTTCTTGT
TNF-α	CCCTCACACTCAGATCATCTTCT	GCTACGACGTGGGCTACAG
IL-17	TTTAACTCCCTTGGCGCAAAA	CTTTCCCTCCGCATTGACAC
IL-23	CAGCAGCTCTCTCGGAATCTC	TGGATACGGGGCACATTATTTTT

### 2.10 Intestinal permeability assay

To assess the integrity of the intestinal barrier, rats were deprived of food and water for 4 h at the end of the experiment, followed by oral administration of FITC–dextran (4 kDa; 600 mg/kg). Four hours after FITC–dextran administration, blood specimens were obtained from the abdominal aorta of anesthetized rats, and serum was isolated. The fluorescence intensity of serum FITC–dextran was measured using a microplate reader at excitation and emission wavelengths of 485 nm and 528 nm, respectively. The level of serum FITC–dextran was assessed by a standard curve and functioned as an indicator of intestinal permeability.

### 2.11 Flow cytometric analysis of immune cell subsets in rat peripheral blood and colonic tissue

For the examination of peripheral blood, 100 μL of blood was combined with 1 μL of antibody and incubated in the dark at ambient temperature for 15 minutes. Subsequently, 2 mL of 1× lysis buffer was introduced into each tube containing 200 μL of whole blood and monoclonal antibody. After adding the lysis buffer, the tubes were gently mixed and incubated in the dark at room temperature for 15 minutes. The samples were subsequently centrifuged at 1,500 rpm for 5 minutes, following which the supernatant was meticulously discarded, the pellet was reconstituted in new PBS, and the specimens underwent a second centrifugation. This washing procedure was repeated until no visible blood cells remained in the pellet. The cells were reconstituted in 0.5 mL of PBS and passed through a 35-μm nylon mesh before flow cytometric evaluation.

For tissue sample analysis, colorectal tissue was excised, thoroughly rinsed with PBS, and transferred to fresh PBS. The tissue was mechanically dissociated through a 200-mesh cell sieve for 6–8 min until a white, mucus-like mass was obtained. The sieve was washed with a minimal amount of PBS, and the resultant cell suspension was filtered and gathered. Subsequently, 1 mL of 1× lysis buffer was added, and the mixture was meticulously stirred and incubated at room temperature for 20 minutes. After incubation, 10 mL of PBS was added, and the blend was carefully agitated. The suspension was centrifuged at 1,500 rpm for 10 minutes, after which the supernatant was removed. The cell pellet was dissolved in 1 mL of PBS, after which the appropriate antibody was introduced. The cells were then positioned in an incubator either on ice or at 4°C for 30 minutes. The cells were washed with 3 mL of PBS by centrifugation at 1,000 rpm for 10 minutes, after which the supernatant was discarded. The cells were finally reassembled in 500 μL of PBS and analyzed by flow cytometry.

For Th17 cell analysis, freshly isolated single-cell suspensions from colonic tissue or peripheral blood mononuclear cells were adjusted to 5 × 10^5^ cells/mL and stimulated with PMA (50 ng/mL) and ionomycin (500 ng/mL) for 4 h. After 2 h of incubation, BFA (1 μL/mL) was added to inhibit cytokine secretion. After stimulation, cells were first surface-stained for CD4 and CD3, then fixed and permeabilized with Foxp3 fixation/permeabilization buffer, and subsequently stained intracellularly with a fluorescently labeled anti-IL-17A antibody. For Treg cell analysis, cell suspensions were first surface-stained for CD4, then fixed and permeabilized with the same reagents, and finally stained intracellularly with a fluorescently labeled anti-Foxp3 antibody.

All flow cytometry data were analyzed using FlowJo software (version 10.9.0). The sequential gating strategy was as follows: debris was first excluded on the FSC-A/SSC-A plot to define the intact cell population; cell aggregates were then excluded on the FSC-A/FSC-H plot; dead cells were subsequently excluded using the live/dead viability dye channel; and CD45^+^ leukocytes were gated on the CD45/SSC plot. Within the gated population, CD4^+^ T cells were used as the parent population to identify CD4^+^IL-17A^+^ Th17 cells and CD4^+^Foxp3^+^ Treg cells, respectively.

### 2.12 Western blot analysis of protein expression in colonic tissue

Total protein was extracted from colonic tissues by homogenization in pre-cooled RIPA lysis buffer. Protein concentrations were determined using the BCA assay, and the total protein loading amount was standardized to 30 μg per lane. Protein samples were separated by 10% SDS-PAGE and subsequently transferred onto PVDF membranes (0.45 μm, Millipore) using a wet transfer method. The membranes were blocked with 5% non-fat milk for 1 h at room temperature and then incubated with specific primary antibodies overnight at 4 °C on a shaker. The following day, the membranes were thoroughly washed with TBST and incubated with the corresponding HRP-conjugated IgG secondary antibodies at a dilution of 1:5,000 for 1 h at room temperature. Protein bands were visualized using enhanced chemiluminescence (ECL), with the exposure time adjusted according to signal intensity. The grayscale intensities of the protein bands were quantified using ImageJ software.

### 2.13 Immunofluorescence detection of Kv1.3 protein expression in NCM460 cells

NCM460 cells were plated in six-well plates at a density of 5 × 10^4^ cells per well and incubated for 24 hours at 37°C in a humidified 5% CO_2_ environment. The cells were treated with either 0.01% DMSO as the vehicle control or 50 μM CLA. Following treatment, the cells were fixed with 4% paraformaldehyde for 20 minutes at room temperature and then incubated with an anti-Kv1.3 primary antibody (1:2,000) at 4°C overnight. The following day, the cells were acclimated to room temperature, meticulously rinsed with PBS, and incubated with a fluorophore-linked secondary antibody for 60 minutes at room temperature in the absence of light. The nuclei were thereafter counterstained with DAPI (1:100) for 5 minutes. Images were obtained by confocal laser-scanning microscopy and assessed to determine the intensity of Kv1.3 immunofluorescence.

### 2.14 Molecular docking of CLA with Kv1.3

The structure of human Kv1.3 (PDB ID: 7SSX) was obtained from UniProt. The three-dimensional structure of CLA (PubChem CID: 84029) was downloaded from PubChem. Water molecules were removed using PyMOL. Polar hydrogen atoms and Gasteiger charges were subsequently incorporated via AutoDockTools, and the structures of the protein and ligand were transformed into PDBQT format. A docking grid box measuring 60 × 60 × 60 Å was established at the center of the channel pore region, and semi-flexible molecular docking was performed using AutoDock Vina with an exhaustiveness parameter of 8. The docking conformation with the lowest predicted binding energy was selected, and its hydrogen-bonding and hydrophobic interactions were analyzed and visualized using PyMOL.

### 2.15 Molecular dynamics simulation of the CLA–Kv1.3 complex

Molecular dynamics simulations were performed using the Schrödinger software suite, employing the OPLS force field and an explicit simple point charge (SPC) water model. The system was gradually equilibrated according to the standard Desmond relaxation protocol, which included a Brownian dynamics NVT stage at 10 K and a Langevin NVT stage. The production simulation was conducted in the NPT ensemble at 300 K and 1.01325 bar, with temperature and pressure regulated using the Martyna–Tobias–Klein (MTK) method. A multiple time-step integration scheme of 2 fs/2 fs/6 fs was employed, and the production simulation was conducted for 100 ns.

### 2.16 Statistical analysis

Statistical analyses were performed using IBM SPSS Statistics 22.0, and graphs were generated using GraphPad Prism 9.0. The individual animal was the experimental unit; technical replicates were averaged before analysis. Data are presented as individual values with the mean ± SEM. Normality and homogeneity of variance were assessed using the Shapiro–Wilk and Levene’s tests, respectively. Two groups were compared using a two-tailed unpaired Student’s t-test, whereas multiple groups were analyzed using one-way ANOVA followed by Tukey’s *post hoc* test. Body weight and DAI were analyzed using two-way repeated-measures ANOVA, reporting the treatment, time, and treatment × time effects, with Greenhouse–Geisser and Bonferroni corrections where appropriate. Mean differences and 95% confidence intervals were reported. Adjusted *P* < 0.05 was considered statistically significant. Flow-cytometry results with n = 3 were considered exploratory.

## 3 Results

### 3.1 CLA demonstrated significant anti-inflammatory effects

CLA reduced LPS-triggered inflammatory responses n both *in vitro* and *in vivo* settings and enhanced cell viability *in vitro*. In cellular studies, CLA decreased LPS-induced concentrations of the inflammatory mediators IL-6, TNF-α, and NO in a dose-dependent manner in Caco-2 and NCM460 cells, while enhancing cell viability (**Figures 1A–D**). In the LPS-induced mouse inflammation model, CLA treatment significantly reduced the serum levels of IL-6, TNF-α, and NO in LPS-treated mice (**Figure 1E**). Immunohistochemical examination demonstrated a considerable elevation in TLR4 and TLR9 expression alongside a notable reduction in OCC and ZO-1 levels in colonic tissues from the model group. CLA therapy significantly rectified these modifications (**Figure 1F**).

**Figure 1:**
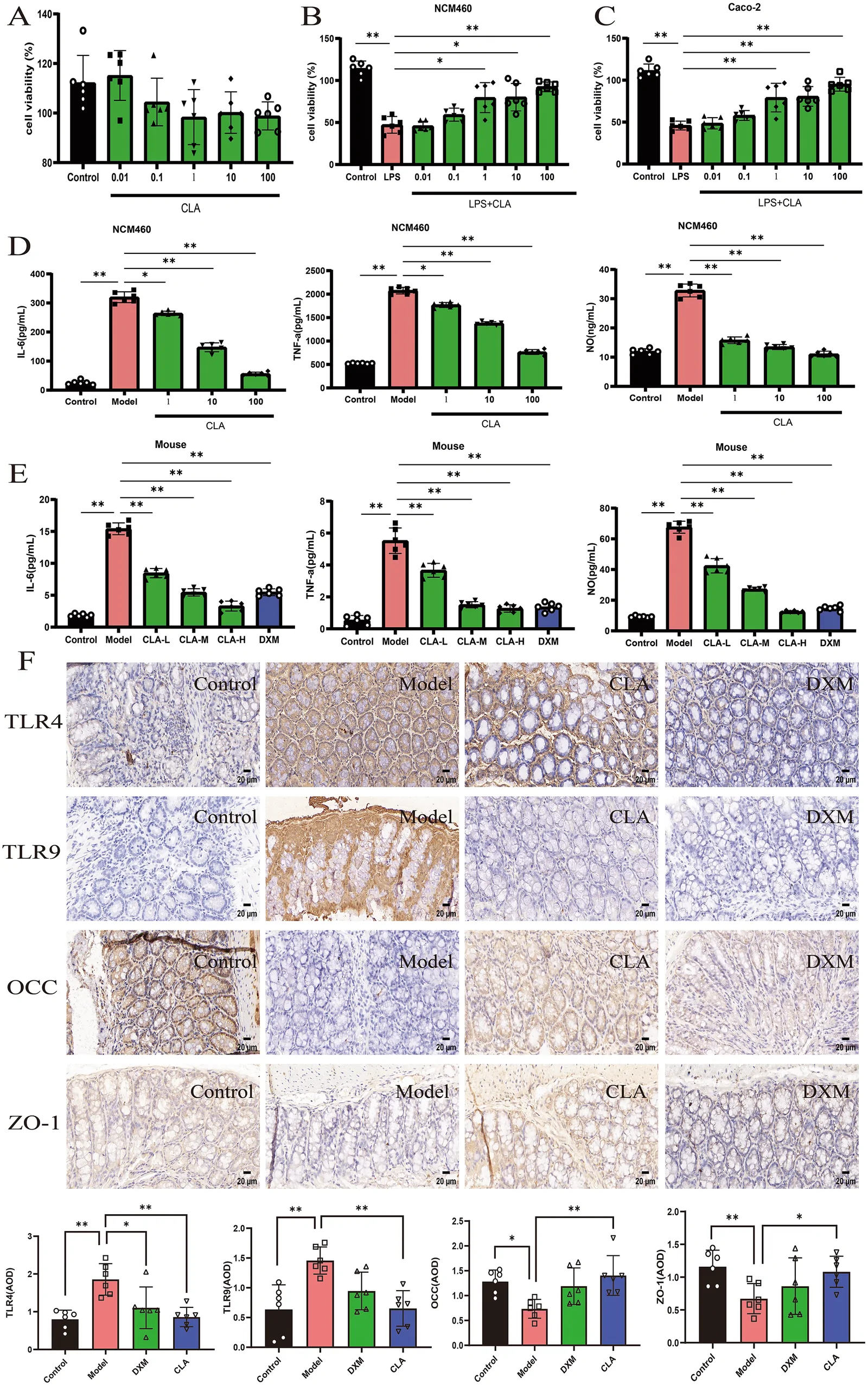
Clarithromycin (CLA) attenuates lipopolysaccharide (LPS)-induced inflammatory responses in vitro and in vivo and improves cell viability *in vitro*. **(A)** Viability of NCM460 human colonic epithelial cells treated with CLA alone (0.01–100 µM) for 24 h. **(B, C)** NCM460 **(B)** and Caco-2 **(C)** cells were exposed to LPS (1 µg/mL) for 24 h and subsequently treated with CLA (0.01–100 µM) for an additional 24 h. Cell viability was measured using the Cell Counting Kit-8 (CCK-8) assay (n = 6). (D) Levels of IL-6, TNF-α, and NO in the culture supernatants of NCM460 cells exposed to LPS (1 µg/mL) for 24 h and subsequently treated with CLA (1, 10, or 100 µM) for 24 h (n = 6). **(E)** Serum levels of IL-6, TNF-α, and NO in C57BL/6J mice receiving LPS and treatment with CLA (2, 4, or 8 mg/kg by oral gavage) or dexamethasone (DXM; 4 mg/kg) for 7 consecutive days (n = 6). **(F)** Representative immunohistochemical staining and average optical density quantification of TLR4, TLR9, occludin (OCC), and ZO-1 in mouse colonic tissue from the control, model, CLA-treated, and DXM-treated groups (scale bar = 20 µm; n = 6). Data are presented as the mean ± SEM. *P < 0.05, **P < 0.01.

### 3.2 CLA alleviated chronic colitis in rats

Throughout the progression of colitis, CLA therapy markedly mitigated DSS-induced weight loss in rats, reduced DAI scores, and reduced the incidence of bloody and loose (**Figures 2A, B**). CLA significantly reduced DSS-induced CMDI. This score is determined based on the degree of mucosal structural damage, the extent of crypt loss, and the degree of inflammatory cell infiltration. It indicates that CLA has a significant protective effect on the intestine (**Figures 2C, D**). CLA treatment significantly reduced intestinal permeability in rats with colitis, suggesting that CLA contributed to the restoration of impaired intestinal barrier function (**Figure 2E**). RT-qPCR assessment revealed that CLA markedly diminished the mRNA expression levels of IL-1β, IL-6, IFN-γ, IL-12, TNF-α, IL-17A, and IL-23 in colonic tissues (**Figures 2F–L**). Histopathological analysis further supported these findings. H&E-stained sections indicated that rats in the CLA-treated cohort displayed significantly diminished disruption of the colonic mucosal architecture, restoration of crypt structure, and a notable decrease in inflammatory cell infiltration (**Figure 2M**). Collectively, CLA effectively alleviated the symptoms of DSS-induced ulcerative colitis in rats by reducing intestinal permeability, suppressing excessive inflammatory responses, and protecting the intestinal epithelial structure.

**Figure 2:**
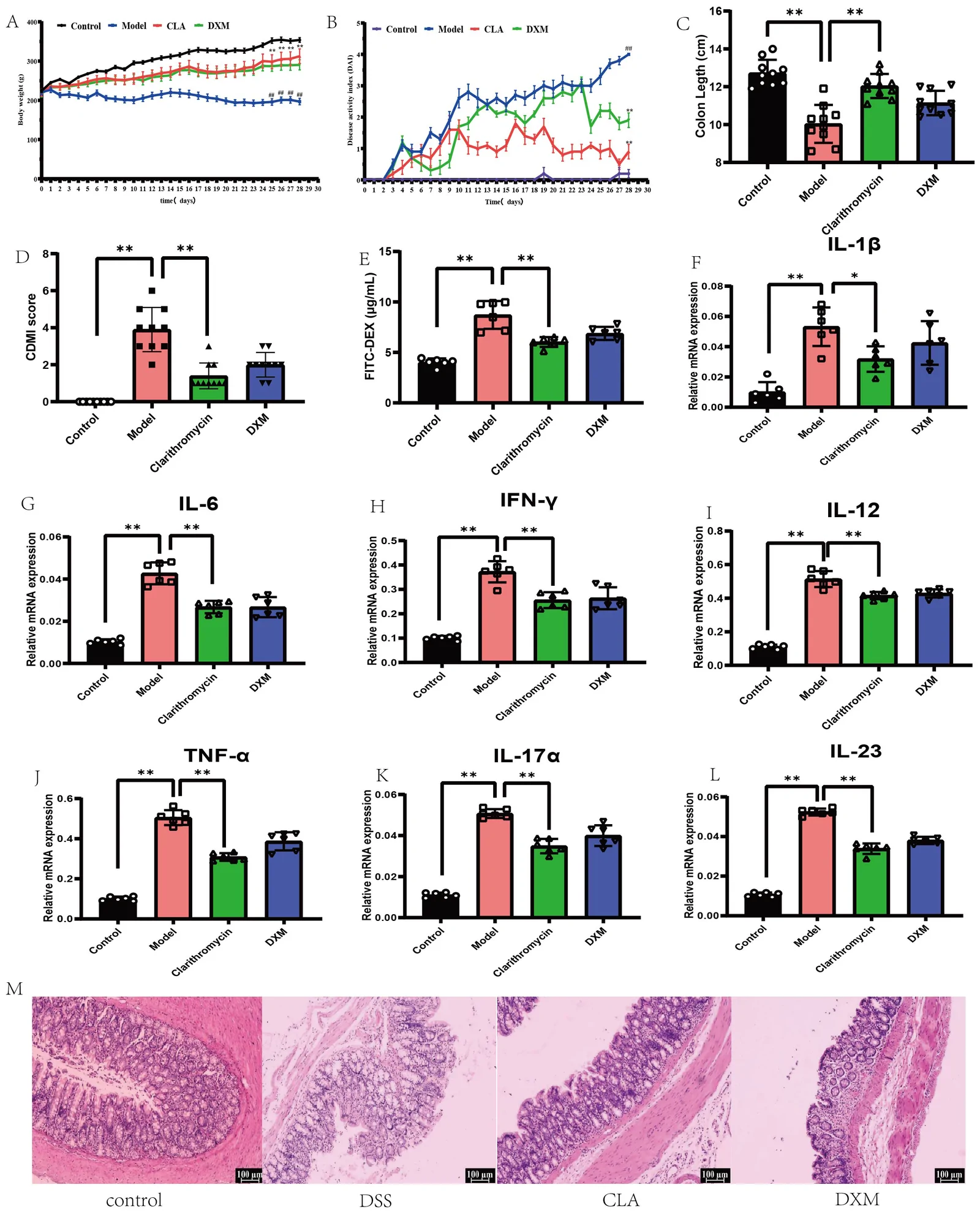
CLA ameliorates dextran sulfate sodium (DSS)-induced chronic colitis in rats and reduces colonic inflammatory cytokine mRNA expression. **(A, B)** Body-weight curves **(A)** and disease activity index (DAI) scores **(B)** during DSS-induced colitis and CLA treatment (n = 10). **(C)** Colon length measurements showing DSS-induced colon shortening and its attenuation by CLA treatment (n = 10). **(D)** Colonic mucosal damage index (CMDI) scores (n = 10). **(E)** Serum FITC–dextran concentrations, used as an indicator of intestinal permeability (n = 6). **(F–L)** Reverse transcription quantitative PCR (RT-qPCR) analysis of the relative mRNA expression levels of interleukin-1β (IL-1β), interleukin-6 (IL-6), interferon-γ (IFN-γ), interleukin-12 (IL-12), tumor necrosis factor-α (TNF-α), interleukin-17A (IL-17A), and interleukin-23 (IL-23) in colonic tissues (n = 6). **(M)** Representative hematoxylin and eosin (H&E)-stained sections showing colonic mucosal architecture, crypt damage, and inflammatory cell infiltration (scale bar = 100 µm; n = 10). Dexamethasone (DXM) served as the positive control. Data are presented as the mean ± SEM. *P < 0.05, **P < 0.01; ##*P* < 0.01, versus the control group.

### 3.3 Effects of CLA on peripheral blood immune cell subsets in rats with DSS-induced colitis

As shown in **Figure 3**, flow cytometric analysis demonstrated that CLA changed the proportions of peripheral blood immune cell subsets in rats with DSS-induced colitis. Following DSS induction, there was a notable increase in the ratios of peripheral blood CD4^+^ (CD3^+^CD4^+^) and CD8^+^ (CD3^+^CD8a^+^) T cells, indicating an exaggerated adaptive immune response. CLA treatment significantly attenuated the abnormal increases in the proportions of both T-cell subsets (**Figures 3A, B**). Similarly, CLA reversed the DSS-induced increase in the proportion of B cells (CD3^-^CD45Ra^+^) (**Figure 3C**), suggesting that CLA suppressed the enhanced humoral immune response. Furthermore, CLA significantly reduced the proportions of NK cells (CD3^-^CD161a^+^) and DCs (CD11b/c^+^CD45^+^) (**Figures 3D, E**). Given the critical antigen-presenting function of DCs, these findings suggest that CLA may attenuate downstream T-cells activation by suppressing DCs function, thereby contributing to the re-establishment of systemic immune homeostasis.

**Figure 3:**
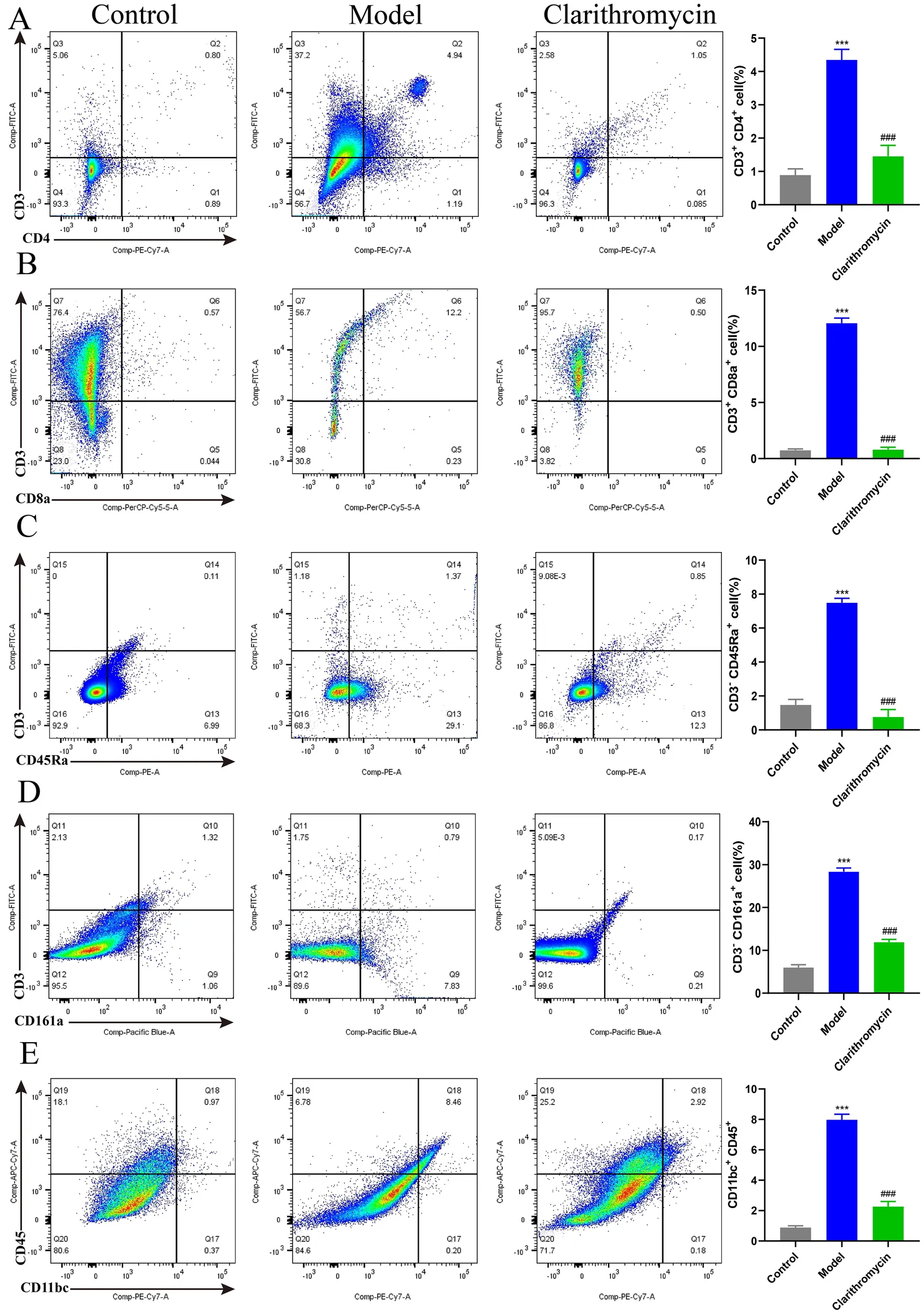
Effects of CLA on peripheral blood immune cell subsets in rats with dextran sulfate sodium (DSS)-induced colitis. The proportions of immune cell subsets in peripheral blood were determined by flow cytometry (n = 3 per group). **(A)** CD4^+^ T cells (CD3^+^CD4^+^); **(B)** CD8^+^ T cells (CD3^+^CD8a^+^); (C) B cells (CD3⁻CD45Ra^+^); **(D)** natural killer (NK) cells (CD3⁻CD161a^+^); and **(E)** dendritic cells (CD11b/c^+^CD45^+^). Data are presented as the mean ± SEM. Differences among groups were analyzed using one-way ANOVA followed by Tukey’s post hoc test. ***P < 0.001, versus the control group; ^###^*P* < 0.001, versus the model group.

### 3.4 Effects of CLA on colonic immune cell subsets in rats with DSS-induced colitis

As illustrated in **Figure 4**, flow cytometric analysis of single-cell suspensions from colonic tissue revealed a significant DSS-induced localized intestinal immune response. The model group exhibited marked infiltration of CD4^+^ and CD8^+^ T cells into the colonic lamina propria. In contrast, CLA treatment significantly reduced the DSS-induced increases in these T cell populations (**Figures 4A, B**), indicating that CLA attenuated T cell-associated injury within the local intestinal microenvironment. At the innate immune level, CLA reversed the abnormal accumulation of B cells and NK cells (**Figures 4C, D**) and significantly reduced DCs infiltration at the intestinal lesion sites (**Figure 4E**). Taken together, the peripheral blood and intestinal data revealed a consistent immunomodulatory pattern following CLA treatment, characterized by reduced DCs infiltration accompanied by decreased expansion and infiltration of effector T cells. These findings suggest that CLA may modulate intestinal immune homeostasis at both systemic and local levels by affecting DCs migration and activation, thereby potentially contributing to its anti-colitic effects.

**Figure 4:**
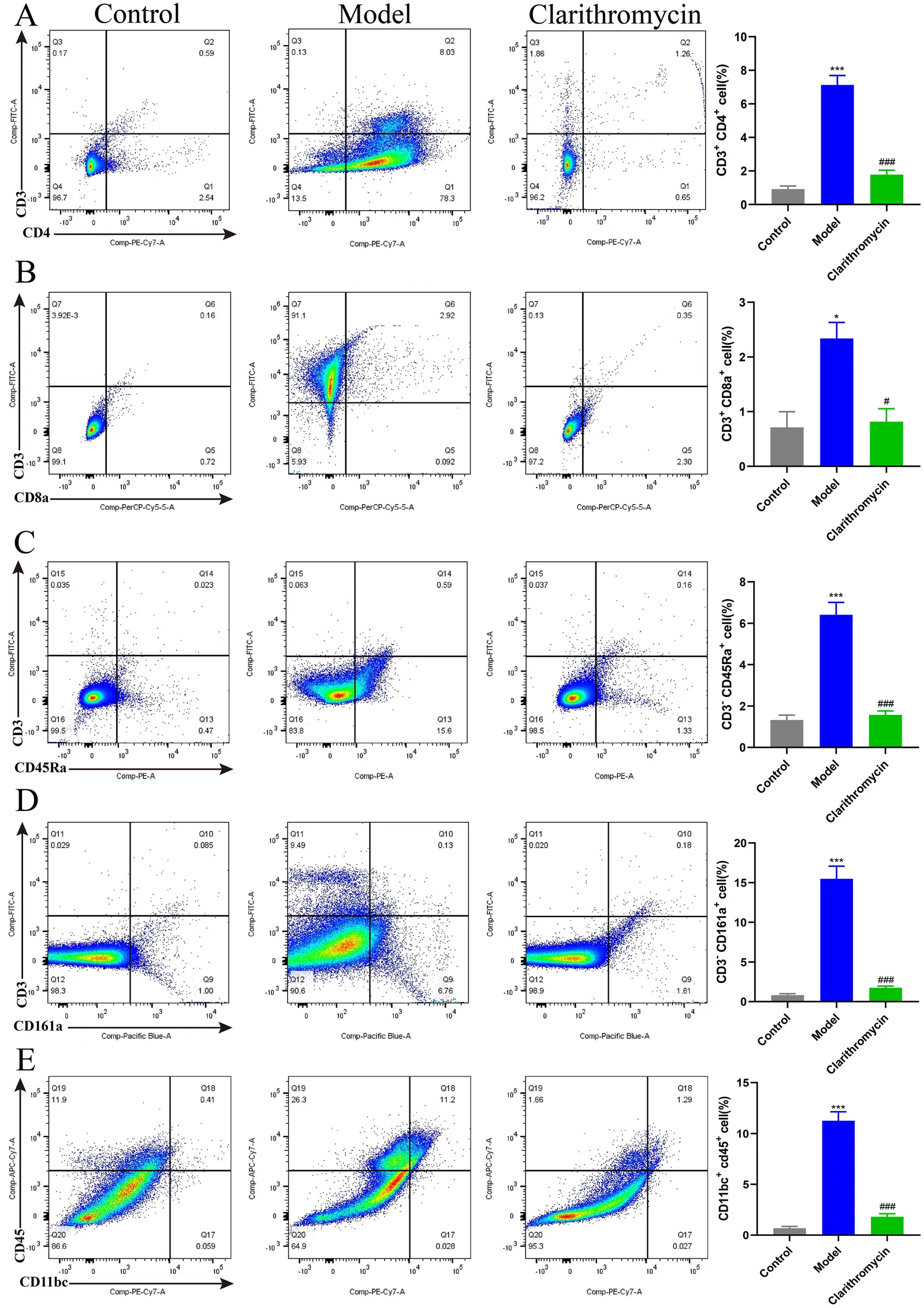
The effect of CLA on the proportions of immune cell subsets in the colon tissues of rats with DSS-induced colitis. The colon tissues were digested to prepare single-cell suspensions, and the proportions of each immune cell subset were quantified by flow cytometry (n = 3). **(A)** CD4^+^ T cells (CD3^+^CD4^+^); **(B)** CD8^+^ T cells (CD3^+^CD8a^+^); **(C)** B cells (CD3^-^CD45Ra^+^); **(D)** NK cells (CD3^-^CD161a^+^); **(E)** Dendritic cells (CD11b/c^+^CD45^+^). Data are presented as the mean ± SEM. *P < 0.05, ***P < 0.001, versus the control group; ^#^*P* < 0.05, ^###^*P* < 0.001, versus the model group.

### 3.5 Effects of CLA on DCs activation in rats with DSS-induced colitis

**Figure 5** demonstrates that flow cytometry was used to assess the effect of CLA on DCs activation status in the peripheral blood and colonic tissue of rats with DSS-induced colitis. In peripheral blood, DSS significantly increased the proportions of CD11b/c^+^MHC-II^+^ and CD11b/c^+^CD80^+^ DCs subsets (**Figures 5A, B**), suggesting increased systemic antigen-presenting activity. Although CLA treatment did not significantly alter the proportion of CD11b/c^+^CD103^+^ DCs (**Figure 5C**), it significantly reduced the proportions of CD11b/c^+^MHC-II^+^ and CD11b/c^+^CD80^+^ DCs subsets, indicating that CLA primarily attenuated the capacity of circulating DCs to initiate adaptive immune responses by suppressing their activated and mature phenotypes.

**Figure 5:**
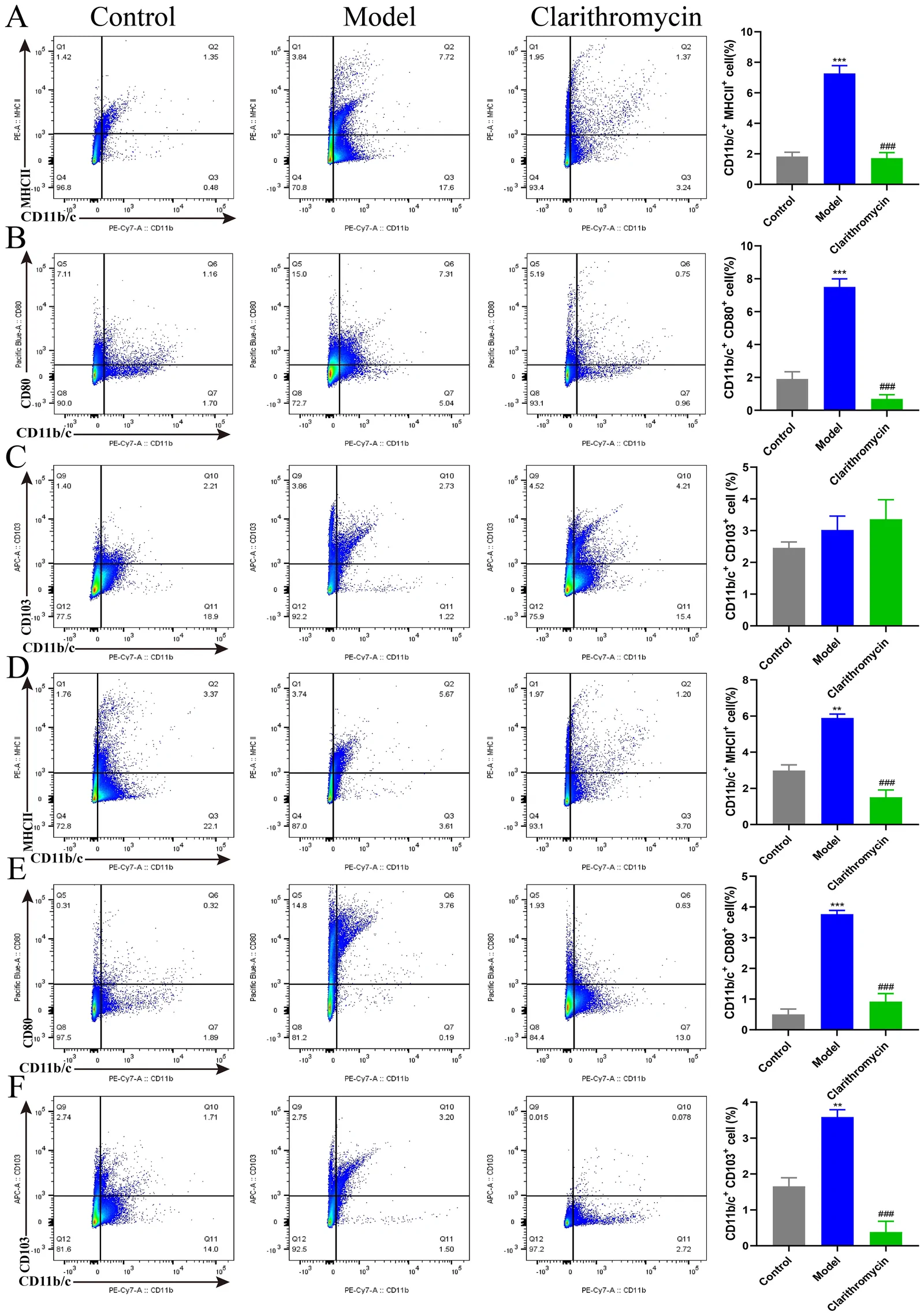
. Effects of clarithromycin (CLA) on dendritic cell phenotypes in the peripheral blood and colonic tissues of rats with dextran sulfate sodium (DSS)-induced colitis. Dendritic cell (DC) subsets in peripheral blood and colonic single-cell suspensions were analyzed by flow cytometry (n = 3 per group). **(A–C)** Proportions of CD11b/c^+^major histocompatibility complex class II (MHC-II)^+^ cells **(A)**, CD11b/c^+^CD80^+^ cells **(B)**, and CD11b/c^+^CD103^+^ cells **(C)** in peripheral blood. **(D–F)** Proportions of CD11b/c^+^MHC-II^+^ cells **(D)**, CD11b/c^+^CD80^+^ cells **(E)**, and CD11b/c^+^CD103^+^ cells **(F)** in colonic tissues. Data are presented as the mean ± SEM. **P < 0.01, ***P < 0.001, versus the control group; ^###^*P* < 0.001, versus the model group.

Conversely, in colonic tissue, DSS administration markedly elevated the ratios of CD11b/c^+^MHC-II^+^ and CD11b/c^+^CD80^+^ DCs subsets (**Figures 5D, E**), along with a notable increase in the proportion of CD11b/c^+^CD103^+^ DCs (**Figure 5F**), consistent with enhanced DCs homing to the intestinal mucosa. CLA treatment significantly reversed the DSS-induced increases in these DCs subsets and markedly reduced the proportion of CD11b/c^+^CD103^+^ DCs with tissue-resident characteristics. Taken together, the effects of CLA on DCs exhibited tissue-dependent differences. In peripheral blood, CLA was primarily associated with reduced DCs activation and maturation. In contrast, at intestinal inflammatory sites, it was associated with suppression of the antigen-presenting phenotype, reduced expression of costimulatory molecules, and decreased mucosal homing of DCs. These findings suggest that CLA may attenuate downstream effector T-cell activation by limiting excessive DCs activation and intestinal recruitment, thereby potentially contributing to its anti-colitic effects.

### 3.6 Effects of CLA on the Th17/Treg balance in rats with DSS-induced colitis

As illustrated in **Figure 6**, DSS significantly elevated the percentage of Th17 cells (CD4^+^IL-17^+^) while diminishing the percentage of Treg cells (CD4^+^Foxp3^+^) in the peripheral blood of rats afflicted with DSS-induced colitis, leading to a notable increase in the Th17/Treg ratio and suggesting an impairment in systemic immunity (**Figures 6A, B**). Following CLA administration, the percentage of peripheral blood Th17 cells was markedly reduced, while the percentage of Treg cells was significantly restored, suggesting that CLA effectively rectified the DSS-induced peripheral Th17/Treg imbalance and mitigated the heightened systemic immune response.

**Figure 6:**
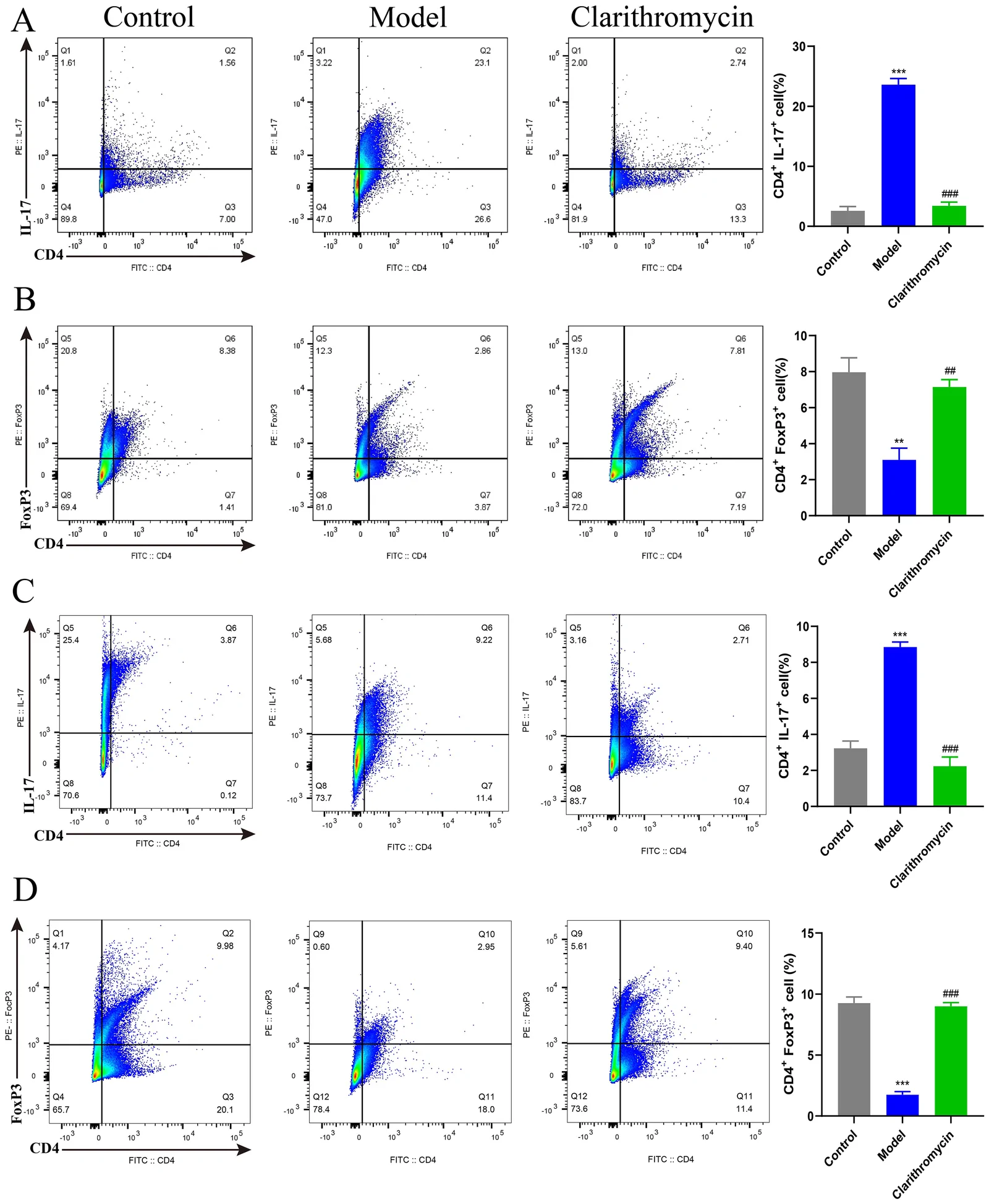
The effect of CLA on the proportions of Th17 and Treg cells in the peripheral blood and colon tissues of rats with colitis induced by DSS. **(A–B)** Flow cytometry was used to detect the proportions of Th17 and Treg cells in the peripheral blood (n = 3); **(A)** Th17 cells (CD4^+^IL-17^+^); **(B)** Treg cells (CD4^+^Foxp3^+^). **(C–D)** Flow cytometry was used to detect the proportions of Th17 and Treg cells in the colon tissues (n = 3); **(C)** Th17 cells (CD4^+^IL-17^+^); **(D)** Treg cells (CD4^+^Foxp3^+^). Data are presented as the mean ± SEM. **P < 0.01, ***P < 0.001, versus the control group; ^##^*P* < 0.01, ^###^*P* < 0.001, versus the model group.

Similarly, DSS administration increased the percentage of Th17 cells and markedly decreased the percentage of Treg cells in colonic tissue (**Figures 6C, D**). Notably, CLA exerted a more pronounced corrective effect on the local intestinal immune imbalance. It not only more efficiently obstructed the ingress of pathogenic Th17 cells into the intestinal mucosa but also significantly increased the percentage of Treg cells with immunosuppressive capabilities. Taken together, CLA reciprocally modulated the proportions of Th17 and Treg cells and restored the Th17/Treg balance at both the systemic and intestinal levels. This immunomodulatory pattern was consistent with the observed suppression of excessive inflammatory responses, maintenance of intestinal immune homeostasis, and alleviation of colitis-associated manifestations following CLA treatment, suggesting that modulation of the Th17/Treg balance may be an important immunoregulatory component of CLA’s anti-colitic effects.

### 3.7 CLA suppresses Kv1.3 protein expression and shows a potential interaction with Kv1.3

Immunofluorescence analysis showed that Kv1.3 fluorescence intensity was markedly increased in LPS-stimulated NCM460 cells compared with control cells, whereas CLA treatment markedly reduced the fluorescence signal (**Figure 7A**). Quantitative Western blot analysis showed a significant increase in the relative expression of Kv1.3 protein in both colonic tissue and isolated T lymphocytes from the experimental group. In contrast, CLA therapy notably reduced Kv1.3 protein expression (**Figures 7B, C**). *In vitro* studies demonstrated that CLA decreased Kv1.3 expression in NCM460 cells in a concentration-dependent manner, with the most pronounced decrease observed at 100 μM (**Figure 7D**).

**Figure 7:**
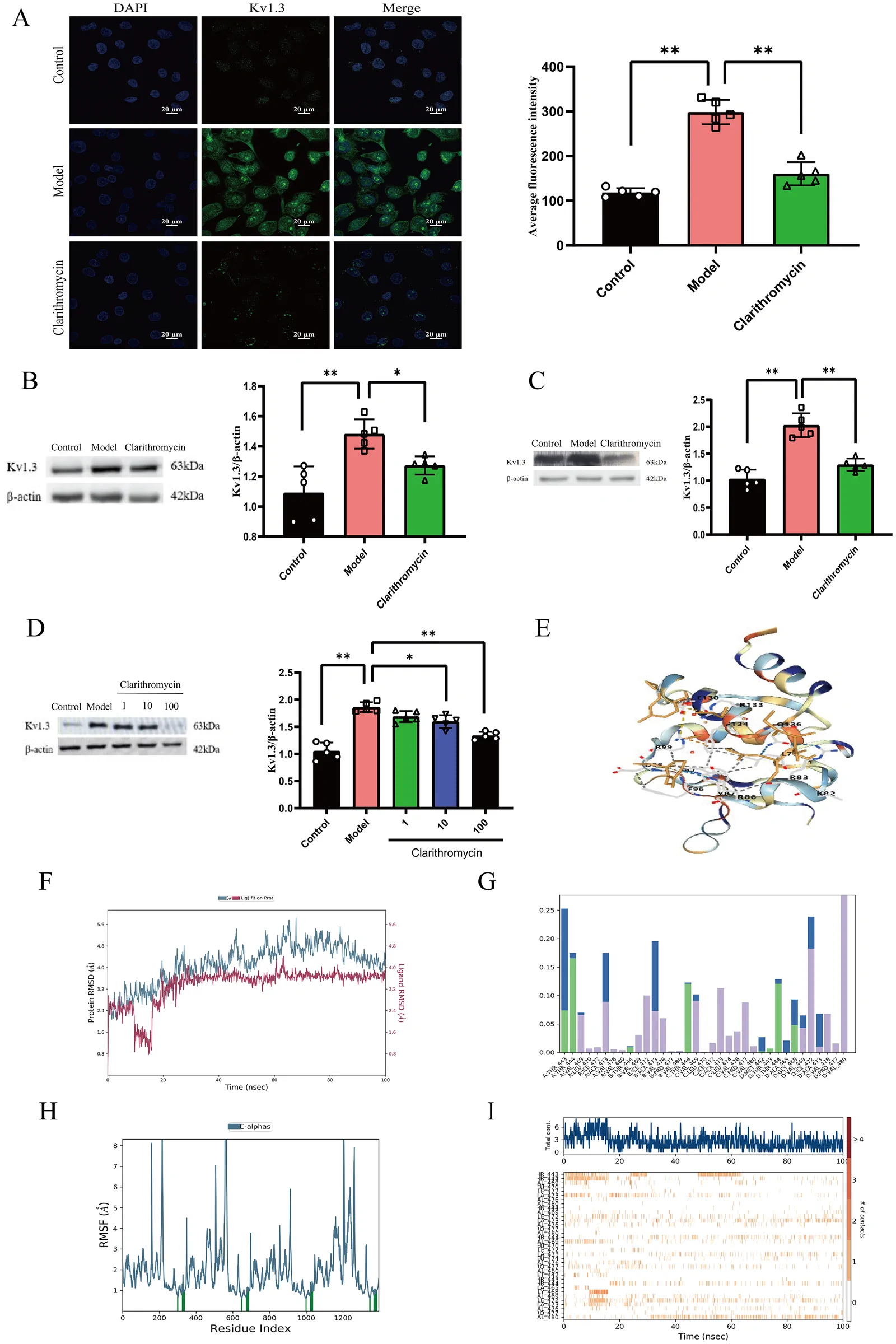
Effect of clarithromycin on Kv1.3 expression. **(A)** Fluorescence intensity of Kv1.3 detected by laser confocal microscopy (scale bar: 20 µm) (n = 5). **(B)** Western blot quantitative analysis of Kv1.3 protein in colonic tissue (n = 5). **(C)** Western blot quantitative analysis of Kv1.3 protein in T cells from colonic tissue (n = 5). **(D)** Western blot quantitative analysis of Kv1.3 protein in NCM460 cells (n = 5). **(E)** Molecular docking diagram of clarithromycin with the Kv1.3 channel protein. **(F)** RMSD profiles of the Kv1.3 protein and CLA ligand during the 100 ns molecular dynamics simulation. **(G)** Interaction fractions between CLA and individual Kv1.3 residues throughout the simulation. **(H)** Root mean square fluctuation (RMSF) analysis of the Cα atoms of individual Kv1.3 residues. **(I)** Total number of CLA–Kv1.3 contacts and the temporal distribution of contacts with individual residues during the 100 ns simulation. Data were expressed as mean ± SEM. *P < 0.05, **P < 0.01.

Molecular docking and molecular dynamics (MD) simulations were performed to evaluate the potential binding mode of CLA with Kv1.3 and characterize the dynamic behavior of the complex. CLA was processed using LigPrep before docking, and the selected ligand state had a net charge of 0. The resulting docking pose yielded a docking score of −10.3 kcal/mol. Subsequently, a 100-ns MD simulation was performed using Desmond under the NPT ensemble at 300 K and 1.01325 bar. The simulation system contained 37,792 water molecules, 4 K^+^ ions, and 4 Cl^-^ ions. Molecular docking showed that CLA occupied the binding pocket of Kv1.3 (**Figure 7E**). During the 100-ns MD simulation, the RMSD values of the Kv1.3 protein and CLA ligand remained within relatively stable ranges following the initial equilibration period, indicating limited overall conformational deviations over the examined timescale (**Figure 7F**). Residue interaction analysis showed that multiple Kv1.3 residues maintained varying degrees of contact with CLA throughout the simulation (**Figure 7G**). RMSF analysis showed that most Kv1.3 residues exhibited relatively limited conformational fluctuations, whereas several regions displayed higher flexibility (**Figure 7H**). The protein–ligand contact timeline further showed that CLA contacts with multiple Kv1.3 residues recurred throughout the simulation (**Figure 7I**). Taken together, these findings indicate that CLA significantly reduced Kv1.3 protein expression in colonic tissues, T cells, and intestinal epithelial cells. Molecular docking and MD simulations further suggested potential binding of CLA within the Kv1.3 binding pocket and the formation of a relatively stable CLA–Kv1.3 complex. These findings provide expression-level and computational evidence supporting an association between CLA and Kv1.3; however, whether CLA directly inhibits Kv1.3 channel function requires further validation using electrophysiological approaches, such as patch-clamp recording.

## 4 Discussion

UC is a persistent and relapsing inflammatory bowel disorder with a complex, multifaceted etiology that includes genetic predisposition, environmental factors, imbalances in gut flora, and dysregulation of the host immune system (11). Although current treatments, including aminosalicylates, glucocorticoids, and biologics, can provide clinical benefit, their use remains limited by a slow onset of action, drug resistance, and serious adverse effects (12). Consequently, the discovery of innovative therapeutic targets and efficacious medicines with little toxicity is of significant clinical relevance. This study systematically investigated the therapeutic effects and potential mechanisms of the macrolide antibiotic CLA in a DSS-induced colitis model. Our findings showed that CLA not only significantly ameliorated the clinical manifestations and histopathological injury associated with colitis but was also accompanied by reduced Kv1.3 expression in colonic tissue and T cells, suppressed DCs maturation and intestinal homing, and restoration of the Th17/Treg balance. These findings suggest that CLA may contribute to the restoration of intestinal immune homeostasis through a regulatory cascade involving reduced Kv1.3 expression, suppression of excessive DCs activation, and subsequent modulation of the Th17/Treg balance. However, a causal relationship between CLA and direct modulation of Kv1.3 channel function remains to be established.

In the present study, CLA exhibited pronounced anti-inflammatory effects in both *in vitro* and *in vivo* settings. In both LPS-induced cellular and murine models of inflammation, CLA significantly reduced the levels of the pro-inflammatory mediators IL-6, TNF-α, and NO. This discovery aligns with earlier studies indicating that macrolide antibiotics can directly inhibit inflammatory responses via antibacterial-independent molecular pathways (13–15). However, unlike traditional nonsteroidal anti-inflammatory drugs (NSAIDs), which act primarily through nonselective cyclooxygenase inhibition, CLA appears to exert its anti-inflammatory effects through multiple molecular targets. In the DSS-induced rat model of chronic colitis, CLA markedly attenuated body weight loss and the DSS-induced elevation in DAI scores while simultaneously improving colonic shortening and mucosal injury, as indicated by lower CMDI values. Notably, CLA significantly reduced intestinal permeability in rats with colitis. Impaired intestinal barrier function is considered an important initiating factor in UC pathogenesis. Increased intestinal permeability facilitates the translocation of bacterial products, such as LPS, thereby sustaining activation of the innate immune system (16, 17). The ability of CLA to restore the damaged intestinal barrier suggests that its mechanism of action may extend beyond direct anti-inflammatory effects to include physical reinforcement of the epithelial barrier. This effect was further supported by histopathological analysis, which showed that colonic crypt architecture was well preserved and inflammatory cell infiltration was significantly reduced in CLA-treated rats. These findings indicate that CLA promoted tissue repair while alleviating inflammation, an effect that may be crucial for preventing UC recurrence.

Hyperactivation of the immune system is a fundamental characteristic of ulcerative colitis. This research employed flow cytometry to comprehensively delineate the immune cell landscape, uncovering a unique immunoregulatory pattern associated with CLA therapy. DSS induction elevated the percentages of CD4^+^ and CD8^+^ T cells, B cells, natural killer (NK) cells, and DCs in both peripheral blood and the colonic lamina propria. CLA therapy markedly reduced the atypical elevations in these immune cell types. Among these immune cell populations, DCs are particularly important because they bridge innate and adaptive immunity. Our data further indicated that the effects of CLA on DCs differed between peripheral blood and the intestinal compartment. In peripheral blood, CLA primarily suppressed DCs maturation, as reflected by reduced expression of MHC-II and the costimulatory molecule CD80, suggesting that CLA may attenuate DCs’ capacity to prime naïve T cells. In the colonic compartment, CLA not only suppressed the activated DCs phenotype but also significantly reduced the proportion of CD103^+^ DCs. CD103^+^ DCs represent a specialized subset with tissue-resident and migratory properties, transporting antigens from the intestine to draining lymph nodes and promoting Treg differentiation in the presence of retinoic acid (RA) (18, 19). The marked suppression of CD103^+^ DCs by CLA in the intestinal compartment suggests that CLA may reduce antigen presentation and immune-activating signals at sites of inflammation, thereby limiting the local infiltration of effector T cells. Such an “upstream” intervention targeting DCs may be more critical than directly suppressing T cells. However, the causal relationship between alterations in DCs phenotypes and the reduction in T-cell infiltration remains to be established and should be further investigated using co-culture systems of DCs and naïve CD4^+^ T cells, together with Kv1.3 loss-of-function experiments.

Given the changes observed in DCs activity, we next evaluated whether CLA influenced T-lymphocyte subsets, focusing specifically on Th17/Treg homeostasis. Disruption of this equilibrium represents an important immunological contributor to the pathogenesis of UC. Th17 cells facilitate neutrophil accumulation and amplify inflammation through effector cytokines such as IL-17A, while IL-23 signaling supports Th17 cell responses. In contrast, Treg cells produce TGF-β and IL-10, which restrain inflammatory responses and thereby preserve self-tolerance (20, 21). Our investigation revealed that in rats administered DSS, the percentage of Th17 cells was markedly elevated in both peripheral blood and colonic tissue. Conversely, the proportion of Treg cells diminished, leading to a significant modification in the Th17/Treg ratio. Following CLA treatment, the proportion of Th17 cells was significantly reduced, whereas the proportion of Treg cells was markedly restored, with this regulatory effect being particularly pronounced in colonic tissues. These findings suggest that the modulation of the Th17/Treg balance by CLA may be associated with its suppression of aberrant DCs activation. Given that DCs-derived IL-6 and TGF-β are key factors involved in Th17 differentiation, whereas retinoic acid metabolites contribute to the induction of Treg cells, modulation of the DCs phenotype by CLA may represent an important link through which CLA influences the Th17/Treg balance. Accordingly, we propose that CLA may contribute to the restoration of the Th17/Treg balance by limiting excessive dendritic cell activation and homing, thereby reducing antigen presentation and pro-inflammatory cytokine signaling. This immunomodulatory effect is consistent with the observed attenuation of excessive inflammatory responses at both the intestinal and systemic levels. However, the causal relationship among DCs modulation, restoration of the Th17/Treg balance, and the therapeutic effects of CLA remains to be established and should be further investigated using co-culture systems of DCs and naïve CD4^+^ T cells, together with Kv1.3 loss-of-function experiments.

To further elucidate the molecular targets underlying the biological effects of CLA described above, we focused on the voltage-gated potassium channel Kv1.3. Kv1.3 is highly expressed in effector memory T cells (TEM), macrophages, and DCs, and plays a critical role in regulating membrane potential, Ca²^+^ signaling, and immune-cell activation and proliferation (22–24). In recent years, Kv1.3 has emerged as an attractive therapeutic target for autoimmune diseases, and selective Kv1.3 inhibitors have shown promising efficacy in experimental models of multiple sclerosis, rheumatoid arthritis, and other immune-mediated disorders (25–27). In the present study, CLA was found to modulate Kv1.3 expression. Immunofluorescence analysis showed that LPS stimulation markedly increased Kv1.3 protein expression in NCM460 cells. Consistently, Western blot analysis demonstrated significantly elevated Kv1.3 protein levels in both the DSS-induced colitis model and LPS-stimulated NCM460 cells, whereas CLA treatment reduced Kv1.3 expression in a dose-dependent manner. Molecular docking and molecular dynamics simulations further suggested a potential interaction between CLA and a putative binding pocket within Kv1.3. Collectively, these findings link the anti-inflammatory effects of CLA to the modulation of an ion channel-associated pathway and provide expression-level evidence supporting Kv1.3 as a candidate therapeutic target for UC. However, whether CLA exerts its therapeutic effects through direct inhibition of Kv1.3 channel function remains to be established. Further studies using patch-clamp electrophysiology, together with Kv1.3 loss-of-function and rescue experiments, are required to determine the causal contribution of Kv1.3 to the pharmacological effects of CLA.

Following an initial period of conformational adjustment, the RMSD values of the Kv1.3 protein and CLA ligand remained within relatively stable ranges during the later stages of the 100 ns MD simulation, indicating limited overall conformational deviations over the timescale examined. Residue interaction analysis showed that multiple Kv1.3 residues maintained contacts with CLA to varying extents throughout the simulation. RMSF analysis indicated relatively limited fluctuations for most Kv1.3 residues, although several regions exhibited greater flexibility. Protein–ligand contact timeline analysis further revealed recurrent contacts between CLA and multiple Kv1.3 residues during the simulation. These computational findings suggest that CLA may interact with specific sites within Kv1.3; however, whether it can inhibit channel function as an allosteric modulator or a direct channel blocker remains to be determined by electrophysiological experiments. Given that Kv1.3 channel activity is required for activation of the calcineurin–NFAT and NF-κB signaling pathways (28), inhibition of Kv1.3 by CLA could impair Ca²^+^ influx in DCs and T cells and thereby attenuate the transcriptional activation of cytokines such as IL-2 and IL-17. This putative mechanism may partly account for the concomitant suppression of DCs activation and modulation of T-cells differentiation observed following CLA treatment in the present study. Previous electrophysiological studies have shown that CLA directly inhibits Kv1.3 currents in mouse thymocytes in a dose-dependent manner, which is consistent with our observations that CLA reduced Kv1.3 protein expression, suppressed DCs activation, and modulated the Th17/Treg balance. The present study, therefore, provides expression-level and computational evidence supporting a potential interaction between CLA and Kv1.3 and offers a basis for understanding the possible structural basis of macrolide-mediated immunomodulation. Nevertheless, a causal relationship between CLA and direct functional inhibition of Kv1.3 remains to be established through patch-clamp recordings and loss-of-function experiments.

As a broad-spectrum macrolide antibiotic, CLA may alter the gut microbiota, representing an unavoidable potential confounding factor in the present study. Because microbiome analyses were not performed, we cannot exclude a contribution of microbiota-mediated effects to the anti-colitic activity of CLA. Nevertheless, several lines of existing evidence suggest that the anti-inflammatory effects of CLA are not entirely dependent on alterations in the gut microbiota. In a drug-repurposing study, Lloyd et al. (29) reported that CLA suppressed NF-κB signaling in microbiota-independent experimental systems, including mouse peritoneal macrophages and intestinal organoids, and reduced disease severity in an LPS-induced acute inflammatory model. These findings support the possibility of direct immunomodulatory effects that do not require changes in the gut microbiota. A randomized controlled trial by Zhao et al. (30) further showed that clarithromycin-containing bismuth quadruple therapy was associated with an immediate reduction in microbial diversity and alterations in community composition.

In contrast, these parameters returned to baseline by week 10. If alterations in the microbiota entirely mediated the anti-inflammatory effects of CLA, these effects might be expected to diminish as the microbiota returned toward baseline. The reported persistence of anti-inflammatory benefits, therefore, is not readily explained by a purely microbiota-dependent mechanism. Notably, Kv1.3, the primary molecular target investigated in this study, is also of independent immunological relevance. In patients with active UC, Kv1.3 is highly expressed in CD4^+^ and CD8^+^ T cells within inflamed colonic mucosa, with its mRNA levels showing significant correlations with TNF-α (R² = 0.61) and IL-17A (R² = 0.51). Pharmacological blockade of Kv1.3 *in vitro* has also been shown to directly reduce the production of IFN-γ, TNF-α, and IL-17A. These observations support an immune cell–intrinsic pathway that does not directly depend on microbiota composition. Nevertheless, we acknowledge that microbiota-related effects may still contribute to the overall therapeutic efficacy of CLA in the complex *in vivo* setting. Future studies using germ-free mice or DSS models following antibiotic-mediated microbiota depletion, combined with 16S rRNA sequencing, may help quantify the relative contributions of microbiota-dependent and microbiota-independent effects.

Several limitations of the present study should be acknowledged. First, although molecular docking and molecular dynamics simulations suggested a potential interaction between CLA and Kv1.3, the present study did not directly assess the effects of CLA on Kv1.3 currents using patch-clamp or other electrophysiological approaches. Therefore, whether CLA directly inhibits Kv1.3 channel function remains undetermined. Second, the present study primarily employed chemically induced acute and chronic colitis models and did not include genetic or chronic relapsing models, which may not fully recapitulate the pathological heterogeneity of human UC. Moreover, the therapeutic effects of CLA in Kv1.3^-/-^ mice or Kv1.3-knockdown models remain to be determined. Third, the gut microbiota was not analyzed, and the contribution of microbiota-mediated effects to the anti-inflammatory activity of CLA cannot therefore be excluded. Fourth, some flow cytometric analyses were based on a relatively small number of biological replicates (n = 3), which limited the statistical power and the precision of effect estimates. The small sample size also limited the robustness of the assessment of distributional assumptions and increased the uncertainty associated with statistical inference. Furthermore, because multiple immune cell populations and outcome measures were evaluated, the potential impact of multiplicity on the type I error rate should be considered. Therefore, these findings should be regarded as exploratory and interpreted with caution. Future studies incorporating larger independent sample sizes, prespecified strategies for multiple-comparison control, and comprehensive reporting of effect sizes and confidence intervals, together with patch-clamp recordings, Kv1.3 loss-of-function and rescue experiments, and germ-free or antibiotic-mediated microbiota-depletion models combined with 16S rRNA sequencing, will be important for confirming these observations and further defining the relative contributions of Kv1.3-dependent and microbiota-dependent mechanisms to the therapeutic effects of CLA.

## 5 Conclusion

In conclusion, this study demonstrates the potential therapeutic value of CLA in ulcerative colitis. CLA reduced intestinal permeability, preserved intestinal epithelial barrier integrity, and was associated with decreased Kv1.3 expression in colonic tissue and T cells, suppressed dendritic cell maturation and intestinal homing, and restoration of the Th17/Treg balance, collectively contributing to anti-inflammatory and immunomodulatory effects at both systemic and intestinal levels. These findings provide additional experimental evidence for the immunomodulatory actions of macrolides and expression-level support for the potential relevance of Kv1.3 as a therapeutic target in UC. Given the pharmacokinetic challenges associated with developing Kv1.3 inhibitors, repurposing CLA, a long-established, clinically used drug, may offer translational value and clinical relevance. Future studies should verify the causal relationship between CLA and direct modulation of Kv1.3 channel function using patch-clamp recordings and Kv1.3 loss-of-function/rescue approaches, and further evaluate the potential role of CLA as an adjunctive therapy for UC in clinical studies.

## Data Availability

The original contributions presented in the study are included in the article/**Supplementary Material**. Further inquiries can be directed to the corresponding author.

## References

[1] Le Berre C Honap S Peyrin-Biroulet L . Ulcerative colitis. Lancet. (2023) 402:571–84. doi: 10.1016/S0140-6736(23)00966-2 37573077

[2] Aslam N Lo S Sikafi R Barnes T Segal J Smith P . A review of the therapeutic management of ulcerative colitis. Ther Adv Gastroenterol. (2022) 15:17562848221138160. doi: 10.1177/17562848221138160 36478780 PMC9720837

[3] Neurath M . Cytokines in inflammatory bowel disease. Nat Rev Immunol. (2014) 14:329–42. doi: 10.1038/nri3661 24751956

[4] Feske S Wulff H Skolnik E . Ion channels in innate and adaptive immunity. Annu Rev Immunol. (2015) 33:291–353. doi: 10.1146/annurev-immunol-032414-112212 25861976 PMC4822408

[5] Chandy K Wulff H Beeton C Pennington M Gutman G Cahalan M . K+ channels as targets for specific immunomodulation. Trends Pharmacol Sci. (2004) 25:280–9. doi: 10.1016/j.tips.2004.03.010 15120495 PMC2749963

[6] Tarcha E Chi V Muñoz-Elías E Bailey D Londono L Upadhyay S . Durable pharmacological responses from the peptide ShK-186, a specific Kv1.3 channel inhibitor that suppresses T cell mediators of autoimmune disease. J Pharmacol Exp Ther. (2012) 342:642–53. doi: 10.1124/jpet.112.191890 22637724 PMC3422530

[7] Kanoh S Rubin B . Mechanisms of action and clinical application of macrolides as immunomodulatory medications. Clin Microbiol Rev. (2010) 23:590–615. doi: 10.1128/CMR.00078-09 20610825 PMC2901655

[8] Spagnolo P Fabbri L Bush A . Long-term macrolide treatment for chronic respiratory disease. Eur Respir J. (2013) 42:239–51. doi: 10.1183/09031936.00136712 23180583

[9] Saiman L Marshall B Mayer-Hamblett N Burns J Quittner A Cibene D . Azithromycin in patients with cystic fibrosis chronically infected with Pseudomonas aeruginosa: a randomized controlled trial. JAMA. (2003) 290:1749–56. doi: 10.1001/jama.290.13.1749 14519709

[10] Kazama I Maruyama Y . Differential effects of clarithromycin and azithromycin on delayed rectifier K (+)-channel currents in murine thymocytes. Pharm Biol. (2013) 51:760–5. doi: 10.3109/13880209.2013.764539 23527897

[11] Qiu P Ishimoto T Fu L Zhang J Zhang Z Liu Y . The gut microbiota in inflammatory bowel disease. Front Cell Infect Microbiol. (2022) 12:733992. doi: 10.3389/fcimb.2022.733992 35273921 PMC8902753

[12] Lasa J Olivera P Danese S Peyrin-Biroulet L . Efficacy and safety of biologics and small molecule drugs for patients with moderate-to-severe ulcerative colitis: a systematic review and network meta-analysis. Lancet Gastroenterol Hepatol. (2022) 7:161–70. doi: 10.1016/S2468-1253(21)00377-0 34856198

[13] Zimmermann P Ziesenitz V Curtis N Ritz N . The immunomodulatory effects of macrolides-a systematic review of the underlying mechanisms. Front Immunol. (2018) 9:302. doi: 10.3389/fimmu.2018.00302 29593707 PMC5859047

[14] Kohri K Tamaoki J Kondo M Aoshiba K Tagaya E Nagai A . Macrolide antibiotics inhibit nitric oxide generation by rat pulmonary alveolar macrophages. Eur Respir J. (2000) 15:62–7. doi: 10.1183/09031936.00.15106200 10678622

[15] Reijnders T Saris A Schultz M van der Poll T . Immunomodulation by macrolides: therapeutic potential for critical care. Lancet Respir Med. (2020) 8:619–30. doi: 10.1016/S2213-2600(20)30080-1 32526189

[16] Martini E Krug S Siegmund B Neurath M Becker C . Mend your fences: The epithelial barrier and its relationship with mucosal immunity in inflammatory bowel disease. Cell Mol Gastroenterol Hepatol. (2017) 4:33–46. doi: 10.1016/j.jcmgh.2017.03.007 28560287 PMC5439240

[17] Su L Shen L Clayburgh DR Nalle SC Sullivan EA Meddings JB . Targeted epithelial tight junction dysfunction causes immune activation and contributes to development of experimental colitis. Gastroenterology. (2009) 136:551–63. doi: 10.1053/j.gastro.2008.10.081 19027740 PMC2712351

[18] Schulz O Jaensson E Persson EK Liu X Worbs T Agace WW . Intestinal CD103+, but not CX3CR1+, antigen sampling cells migrate in lymph and serve classical dendritic cell functions. J Exp Med. (2009) 206:3101–14. doi: 10.1084/jem.20091925 20008524 PMC2806467

[19] Park K Mikulski Z Seo GY Andreyev AY Marcovecchio P Blatchley A . The transcription factor NR4A3 controls CD103+ dendritic cell migration. J Clin Invest. (2016) 126:4603–15. doi: 10.1172/JCI87081 27820700 PMC5127666

[20] Gaffen S Jain R Garg A Cua D . The IL-23-IL-17 immune axis: from mechanisms to therapeutic testing. Nat Rev Immunol. (2014) 14:585–600. doi: 10.1038/nri3707 25145755 PMC4281037

[21] Josefowicz S Lu L Rudensky A . Regulatory T cells: mechanisms of differentiation and function. Annu Rev Immunol. (2012) 30:531–64. doi: 10.1146/annurev.immunol.25.022106.141623 22224781 PMC6066374

[22] Beeton C Wulff H Standifer NE Azam P Mullen KM Pennington MW . Kv1.3 channels are a therapeutic target for T cell-mediated autoimmune diseases. Proc Natl Acad Sci USA. (2006) 103:17414–9. doi: pnas.241497298/pnas.0605136103. PMID: 17088564 10.1073/pnas.0605136103 PMC1859943

[23] Olivencia MA Martínez-Casales M Peraza DA García-Redondo AB Mondéjar-Parreño G Hernanz R . KV1.3 channels are novel determinants of macrophage-dependent endothelial dysfunction in angiotensin II-induced hypertension in mice. Br J Pharmacol. (2021) 178:1836–54. doi: 10.1111/bph.15407 33556997

[24] Mullen KM Rozycka M Rus H Hu L Cudrici C Zafranskaia E . Potassium channels Kv1.3 and Kv1.5 are expressed on blood-derived dendritic cells in the central nervous system. Ann Neurol. (2006) 60:118–27. doi: 10.1002/ana.20884 16729292

[25] Chandy K Norton R . Peptide blockers of K_v_1.3 channels in T cells as therapeutics for autoimmune disease. Curr Opin Chem Biol. (2017) 38:97–107. doi: 10.1016/j.cbpa.2017.02.015 28412597

[26] Tajti G Wai D Panyi G Norton R . The voltage-gated potassium channel Kv1.3 as a therapeutic target for venom-derived peptides. Biochem Pharmacol. (2020) 181:114146. doi: 10.1016/j.bcp.2020.114146 32653588

[27] Beeton C Wulff H Barbaria J Clot-Faybesse O Pennington M Bernard D . Selective blockade of T lymphocyte K^+^ channels ameliorates experimental autoimmune encephalomyelitis, a model for multiple sclerosis. Proc Natl Acad Sci USA. (2001) 98:13942–7. doi: 10.1073/pnas.241497298 11717451 PMC61146

[28] Sun B Shen K Zhao R Li Y Lin J . Clarithromycin attenuates airway epithelial-mesenchymal transition in ovalbumin-induced asthmatic mice through modulation of Kv1.3 channels and PI3K/Akt signaling. Int Immunopharmacol. (2024) 139:112624. doi: 10.1016/j.intimp.2024.112624 39002519

[29] Lloyd K Papoutsopoulou S Smith E Stegmaier P Bergey F Morris L . Using systems medicine to identify a therapeutic agent with potential for repurposing in inflammatory bowel disease. Dis Model Mech. (2020) 13:dmm044040. doi: 10.1242/dmm.044040 32958515 PMC7710021

[30] Zhao JT Zhang Y Wang XW Zou PY Zhao Z Mei H . Long-term effects of fecal microbiota transplantation on gut microbiota after Helicobacter pylori eradication with bismuth quadruple therapy: A randomized controlled trial. Helicobacter. (2024) 29:e13079. doi: 10.1111/hel.13079 38984661

