# Supplementary material for: Mechanistic investigation of clarithromycin in ulcerative colitis involving Kv1.3 downregulation and modulation of the Th17/Treg balance
Source: Front Immunol. 2026 Oct 20;17:1957785. doi: 10.3389/fimmu.2026.1957785 (PMC13727478; doi:10.3389/fimmu.2026.1957785)
Supplement: Supplementary file 1 [file DataSheet1.pdf]

frontiers

The International Society for the Advancement of Cytometry (ISAC) has highlighted the importance of including comprehensive methodological information to ensure data reproducibility and reliability. In line with this, Frontiers in Immunology now requires authors to submit a checklist for manuscripts that involve flow or mass cytometry. This checklist helps standardize the reporting process, improving the quality and transparency of published data. By doing so, we support scientific progress, making it easier for other researchers to replicate and validate experiments.

This form should be submitted with any manuscripts using flow or mass cytometry.

Sample/specimen/material description

- [x] Total blood
- [ ] PBMCs
- [x] Organ digests

Other______________________________________

Did the samples suffer any treatment before or after incubation with the antibodies?

- [x] Drug Clarithromycin (4 mg/kg), administered by oral gavage once daily for 7 consecutive days beginning on day 1 of each DSS
- [ ] Cell permeabilization ______________________
- [ ] Dye ____________________________________
- [ ] Propidium iodine
- [ ] Not applicable

Other ______________________________________

Instrument and antibodies

Name of the Cytometer__________________________________

Antibodies and targets	Fluorochrome/ Metal	Catalog number/Company
e.g. anti-CD4	FITC	Cat. XXX/ XXX ltd.
anti-CD3	FITC	Thermo Fisher/eBioscience 11-0030-82

anti-CD4	PE-Cy7	BD Pharmingen/561578
CD8a	PerCP-Cy5-5A	BD Pharmingen/558824
CD45Ra	PE	BD Pharmingen/551402
CD161a	Pacific Blue	BD OptiBuild/744049
CD11b/c	PE-Cy7	BD Pharmingen/562222
MHC-II	PE	BD Pharmingen/554929
CD80	Pacific Blue	BD OptiBuild/743863
CD103	APC	Miltenyi Biotec/130-108-210
CD4	FITC	Thermo Fisher/eBioscience
IL-17	PE	Thermo Fisher/eBioscience
FoxP3	PE	Thermo Fisher/eBioscience

Data analyses

1. Name of the software FlowJo_v10.90________________________________
2. Reference gating strategy in the manuscript or supplementary material

Gating strategy in (eg Figure X) Gating strategy in: Methods, Section 2.11, Fourth paragraph.
